# Knowledge, Attitude, and Practice of Voluntary Blood Donation among Healthcare Workers at the University of Benin Teaching Hospital, Benin City, Nigeria

**DOI:** 10.1155/2013/797830

**Published:** 2013-10-09

**Authors:** Benedict Nwogoh, Usimenahon Aigberadion, Alexander Ikenna Nwannadi

**Affiliations:** ^1^Department of Haematology and Blood Transfusion, University of Benin Teaching Hospital, P.M.B 1111, Benin City 300283, Nigeria; ^2^Department of Haematology, Benue State University, Makurdi 102119, Nigeria

## Abstract

*Introduction.* Adequate and safe blood supply has remained a challenge in developing countries like ours. There is a high dependency on family replacement and remunerated blood donors in our environment which carries an attendant increased risk of transfusion transmissible infection. *Objectives.* The objectives of this study were to assess the knowledge, attitude, and practice of voluntary blood donation among healthcare workers (nonphysicians) and to identify and recruit potential voluntary blood donors. *Methodology.* This was a cross-sectional descriptive study carried out at the University of Benin Teaching Hospital, Benin City. A total of 163 staffs were recruited. Pretest questionnaires were used to assess their knowledge, attitude, and practice of voluntary blood donation. *Statistical Analysis.* The responses were collated and analyzed with the Statistical Package for Social Sciences (SPSS) 16. The association between blood donation practice and gender of respondents, category of staff, and level of education was tested using Chi-square and Fisher's tests where appropriate. *P* < 0.05 were considered statistically significant. *Results.* The median age of the respondents was 32 years (18–56) with females accounting for 55.6% (90). A total of 74.8% (122) attained tertiary education, and 55.8% (91) of respondents were senior staffs. The majority has good knowledge and positive attitude towards donation; however, only 22.1% (36) have donated blood with 41.7% (15) of these being voluntary. Male workers were more likely to donate (*P* < 0.05). There is no significant association between blood donation and level of education. *Conclusion.* There is a strong disparity between the knowledge, attitude, and practice of voluntary donation amongst healthcare workers.

## 1. Introduction

In spite of extensive promising research, a true substitute for blood and blood components may not be available for many years [1]. Therefore, blood donation by humans will continue to be the major source for blood and blood components. There are no national data in Nigeria on blood donor demand and supply; however, available data suggest that over 7,000 units of blood are utilized annually at the University of Benin Teaching Hospital, most of which are provided by blood vendors [2, 3]. 

Donated blood can be lifesaving for individuals who have lost large volumes of blood from serious accidents, obstetric and gynecological hemorrhages, or surgery and stem cell transplant patients as well as for individuals who have symptomatic anemia from medical or hematologic conditions or cancers. Therefore, blood is an important concern to the society. The use of these life saving products may be complicated by infectious and immunological diseases some of which could be life threatening.

Blood banks are obligated to provide adequate and safe blood to the community. Generally, donors are classified into the following categories: voluntary, family replacement, remunerated or paid donors, and autologous donor. The safest donors are found among people who donate their blood voluntarily purely out of altruism and are self-aware of their unsuitability to serve as blood donors where there might be a slightest risk of causing health damage for blood recipients [4, 5]. The risk of transfusion transmissible diseases is highest with the use of blood procured from remunerated donors [2, 3, 6–9]. A person in need for money is more likely to conceal his/her true state of health. Monetary remuneration, which is often offered as a donor motivating tool, might be highly appealing for people who live in desperate straits.

In developing countries like Nigeria, there is a dependency on family replacement and remunerated donors [3, 10–12]. Voluntary blood donation accounts for less than 5% of blood procured in most of our blood banks [13]. The World Health Organization advocates that member states should establish national blood transfusion services that will operate on the basis of voluntary, nonremunerable blood donation [14]. Despite the establishment of National Blood Transfusion Service (NBTS) in Nigeria, very little progress has been made in the direction of providing sufficient blood for our teaming populace. 

The attitude, beliefs, and level of knowledge associated with blood donation may affect the disposition of potential donors to blood donation. Health workers are expected to have a good knowledge of blood usage, to be aware of the increasing demand and scarcity of the products, and are thus expected to donate as well as encourage voluntary blood donation among the public. 

## 2. Study Objectives

The objectives of this study were to assess the knowledge, attitude and, practice of voluntary blood donation among healthcare workers (nonphysicians), to identify and recruit potential voluntary blood donors and to determine the association between blood donation, gender, category of staff, and level of education of the staffs.

## 3. Materials and Methods

This was a cross-sectional descriptive study carried out at the University of Benin Teaching Hospital, Benin City. The centre operates a hospital-based blood banking system which is highly dependent on blood procurement from vendors who operate paid-donor outlets. Their supplies are augmented by supply from the National Blood Transfusion Service (NBTS), family replacement, and very few voluntary donors. The hospital has over 3,000 health workers (nonphysicians) in various departments. 

The staffs are categorized as junior or senior staffs based on their academic qualifications and their job descriptions. Senior staffs are employed into positions requiring higher qualifications such as scientists, nurses, engineers, and accountants, while junior staffs are employed into positions which often do not require a university degree qualification such as office assistants, record clerks, and technicians.

Pretested questionnaires were used to assess their knowledge, attitude, and practice of voluntary blood donation. The questionnaire was modeled after that used in our earlier research work on voluntary donations by physicians [15]. 

A total of 250 health workers were recruited from the various departments by quota sampling to participate in the study. 

*Statistical Analysis.* The responses were collated and analyzed with the Statistical Package for Social Sciences (SPSS) 16. The association between blood donation practice and gender of respondents, category of staff, and level of education was tested using Chi-square and Fisher's tests where appropriate. *P* < 0.05  were considered statistically significant.

## 4. Results

### 4.1. Demographic Parameters

A total of 163 health workers responded to the questionnaire giving a response rate of 65.2%. The age range of the respondents was 18–56 years (median age was 32 years). The male to female ratio is 1 : 1.3. Eighty-six (52.8%) were married, while 75 (46%) were singles. Most (74.8% of respondents) attained tertiary education; ninety-one (55.8%) were senior staffs, and 69 (42.3%) were junior staffs. Nursing staffs were 30 (18.4%), laboratory staffs 37 (22.7%), pharmacy staffs 19 (11.7%), and administrative workers 22 (13.5%) among others. Details of their demographic parameters are represented in Table 1.

### 4.2. Knowledge of Blood Transfusion

A total of 151 (92.6%) respondents expressed good knowledge of the common blood group types, and 153 (93.9%) knew their own blood groups. The blood groups of respondent were A Rhesus (Rh) positive (25) (15.3%), AB Rh positive (3) (1.8%), B Rh positive (16) (9.8%), O Rh negative (6) (3.7%), and O Rh positive (74) (45.4%). Thirty-five persons (21.5%) did not respond, and 4 (2.5%) responses were invalid.

Most respondents (157) (95.7%) were aware of the risk of transmission of infection by transfusion. The risk of transmission of HIV, HBV, HCV, and Syphilis was affirmed by 91.4% (149), 69.9% (114), 42.9% (70), and 27.0% (44), respectively. 

Forty-four (27%) stated that the minimum interval between donations is 6 months, 35 (21.5%) said 3 months, and 13 (8.0%) said a month, while 33 (20.2%) said they have no knowledge of this. 

The majority of the respondents had a good knowledge on who should and who should not donate. However, 6 (3.7%) of the respondents said vulnerable group (sex workers, intravenous drug users) should donate. Twelve (7.4%) and 10 (6.1%) respondents said people should not donate for religious and cultural reasons, respectively. 

Only 66 (40.5%) knew the correct volume of blood collected in the process. Similarly, 59 (36.2%) knew that the donation process lasts less than 20 minutes. Details of the respondents knowledge of blood donation are shown in Table 2.

### 4.3. Attitude towards Blood Donation

A hundred and thirty-three (81.6%) respondents said blood donation was good. Voluntary donation was accepted as the best source of blood donors by 116 (71.2%), replacement donors by 11 (6.7%), remunerated by 3 (1.8%), and self donation by 3 (1.8%). 

One hundred and twenty-five (71.2%) said blood donation may have adverse consequences. Twenty (12.3%) said a donor can contract infection, 99 (60.7%) said the donor may experience temporary weakness, and 9 (5.5%) said the donor may fall sick.

One hundred and forty-six (89.6%) feels that patient relatives should be asked to donate, and 149 respondents have asked relatives in the past to donate. Details of respondents' attitude to blood donation are shown in Table 3.

### 4.4. Practice of Blood Donation

Thirty-six (22.1%) have donated in the past. Only 5 (13.9%) were regular donors. Only 15 (41.7%) are voluntary, and 19 (52.8%) donated to a friend or relative in need of blood.

The reasons for nondonation by those who have not donated include nobody approached them for donation (32) (25.2%), unfit to donate (21) (16.5%), need to donate for a friend or relative in future (25) (19.7%), fear of needle (8) (6.3%), fear of knowing their viral status (4) (3.1%), the donated blood may be sold (5) (3.9%), nonremuneration (1) (0.8%), and their religion prohibits blood donation (1) (0.8%). 

Sixty-seven (41.1%) respondents accepted to be invited to donate blood, but only 32 (19.6%) gave their contacts so that they can be reached.

There was a significant association between male gender and blood donation (*P* = 0.003) as shown in Table 4. The level of education and category of staff have no significant association with the practice of blood donation (*P* = 0.895 and 0.334, resp.) as shown in Tables 5 and 6.

## 5. Discussion

The overdependence on family replacement and remunerated donors to meet the increasing demand for blood and blood products poses serious danger to potential recipient. Settings like ours which is still dependent on serological screening test for detection of transfusion transmissible infections in potential donors; who do not use pathogen nucleic acid detection technique; who do not practice pathogen inactivation; who do not leucodeplete nor irradiate blood products are more at risk. Hence, there is need to improve the recruitment and retention of voluntary-donor population to ensure a reasonably safe blood transfusion practice.

There are lots of publications assessing the knowledge, attitude, and practice of voluntary blood donation; however, very few studies have been published which assess the same on the healthcare workers in our environment and globally. This study has shown as expected that healthcare workers have a good knowledge of blood groups, possible transfusion transmissible infection, and the appropriate donor population but on the contrary exhibited a poor knowledge of the blood donation process.

The study also revealed a positive attitude of the workers towards blood donation, but there was a serious contradiction in the practice of voluntary blood donation. Though all study participants are within the age range of potential donors, only 22.1% of them have donated blood in the past, out of which 52.8% were as family replacement and 41.7% voluntary. This is a far cry based on the knowledge and attitude displayed in this study and their experience of the increased demand for blood in the hospital. A total of 67 (41.1%) of the participants accepted to be invited in the future for voluntary donation but only 32 (19.6%) gave their contacts. This reflects the absence of commitment to their acceptance. Thus, there is a serious disparity in their knowledge, attitude, and practice. 

In a similar study conducted on physicians in the same healthcare facility, a similar trend was observed, but higher proportion of donors and regular donors was reported in the physicians [15]. We recorded that 41.4% blood donation was by physicians, with 39.6% being regular donors and 53.4% of these were voluntary [15].

Study by Mullah et al. assessing the knowledge and perception of healthcare support staffs of a tertiary healthcare facility in Gujarat revealed a poor knowledge of donor eligibility among the staffs, 91% of them perceive blood donation as unsafe, and about 39% of them have donated blood [16].

In a similar study in Iran, Reza et al. assessed the knowledge of 122 healthcare workers and found that 51.6% (just above average) have acceptable knowledge on proper methods of blood and components transfusion [17]. Variation in knowledge of blood donation and transfusion processes may be attributable to the degree of enlightenment program available and the degree of involvement of staffs in blood and blood products management.

The major reason given by those who had never donated was that no one approached them to donate. This highlights the need for serious sensitization and education to all and sundry through the mass media to encourage the populace to approach the blood bank for a blood donation exercise.

This study found a significant association between blood donation and sex. Males in our society are more likely to donate blood than females. This is quite understandable since women within the donor age range usually may have one factor or another interfering with their chances of being suitable to donate. Factors such as their frequent menstrual cycles, pregnancy, and lactation may prevent them from donation. This is in affirmation of the WHO report that there are more male donors in Nigeria [18].

In conclusion, healthcare workers are reasonably informed and have positive perception towards blood donation; however, only few of them have donated and are positively disposed to donate blood. There is a need for active education program to encourage all, and sundry if adequate and safe blood will be guaranteed.

## Figures and Tables

**Table 1:** Sociodemographic parameters of respondents.

(1) Age range (median age)	18–56 (32) yrs	

	*N* = 163	Percentage (%)

(2) Gender		
Males	69	42.3
Females	90	55.2
No response	4	2.5
(3) Marital status		
Single	75	46.0
Married	86	52.8
Widow	1	0.6
No response	1	0.6
(4) Educational status		
None	1	0.6
Primary	6	3.7
Secondary	29	17.8
Tertiary	122	74.8
No response	5	3.1
(5) Category of staff		
Junior	69	42.3
Senior	91	55.8
No response	3	1.8
(6) Department		
Nursing service	30	18.4
Administration	22	13.5
Pharmacy	19	11.7
Laboratory (pathology)	37	22.7
Records	10	6.1
Accounts	9	5.5
Dentistry	6	3.7
Engineering	6	3.7
Physiotherapy	9	5.5
Radiology	9	5.5
Others	6	3.7

**Table 2:** Knowledge on blood donation.

Do you know the common blood groups?	
Yes	151 (92.6%)
No	9 (5.5%)
No response	3 (1.8%)
Do you know your blood group?	
Yes	153 (93.9%)
No	7 (4.3%)
No response	3 (1.8%)
Blood group of respondent?	
A Positive	25 (15.3%)
AB Positive	3 (1.8%)
B Positive	16 (9.8%)
O Negative	6 (3.7%)
O Positive	74 (45.4%)
Invalid	4 (2.5%)
No response	35 (21.5%)
Can a person be infected by receiving blood transfusion?	
Yes (correct)	159 (97.5%)
No (incorrect)	2 (1.2%)
No response	2 (1.2%)
What diseases are transmissible by blood transfusion?	
HIV	149 (91.4%)
HBV	114 (69.9%)
HCV	70 (42.9%)
Syphilis	44 (27.0%)
Malaria	7 (4.3%)
No response	5 (3.1%)
How often can an individual donate?	
Weekly	3 (1.8%)
Monthly	13 (8.0%)
3 Monthly	35 (21.5%)
6 Monthly	44 (27%)
Annually	23 (14.1%)
Don't know	33 (20.2%)
Invalid	1 (0.6%)
No response	11 (6.7%)
Who should donate blood?	
Men (correct)	132 (81.0%)
Women (correct)	112 (81.0%)
Young (<18 yrs) (incorrect)	12 (7.4%)
Old (>60 yrs) (incorrect)	4 (2.5%)
Vulnerable group (incorrect)	6 (3.7%)
Healthy (correct)	111 (68.1%)
Diseased (incorrect)	1 (0.6%)
Who should not donate blood?	
Men (incorrect)	7 (4.3%)
Woman (incorrect)	13 (8.0%)
Young (<18 yrs) (correct)	58 (35.6%)
Old (>60 yrs) (correct)	82 (50.3%)
Vulnerable group (correct)	103 (63.2%)
Healthy (incorrect)	4 (2.5%)
Diseased (correct)	107 (65.6%)
Culture belief (incorrect)	10 (6.1%)
Religious belief	12 (7.4%)
No response	7 (4.3%)
What volume of blood is collected during each donation?	
≤500 mls (correct)	66 (40.5%)
500–1000 mls (incorrect)	37 (22.7%)
Don't know	52 (31.9%)
No response	8 (4.9%)
What is the duration of a donation process?	
<20 minutes (correct)	59 (36.2%)
20–60 minutes	43 (26.4%)
Don't know	52 (31.9%)
No response	9 (5.5%)

**Table 3:** Attitude and practice of blood donation.

	*N* (%)
*Section C*: *Attitude towards blood donation *	
What do you think about blood donation?	
Good	133 (81.6)
Bad	8 (4.9)
Neutral	20 (12.3)
No response	2 (1.2)
What do you think is the best source of blood donors?	
Voluntary donor	116 (71.2)
Replacement donor	11 (6.7)
Remunerated donor	3 (1.8)
Self donor	3 (1.8)
I don't know	8 (4.9)
No response	22 (13.5)
Can something harmful happen to a blood donor during or after donation?	
Yes	125 (76.7)
No	30 (18.4)
I don't know	8 (4.9)
What can happen to a blood donor during or after donation?	
Contract Infection	20 (12.3)
Temporary Weakness	99 (60.7)
Fall Sick	9 (5.5)
Should patient relatives be asked to donate?	
Yes	146 (89.6)
No	5 (3.1)
I don't know	5 (3.1)
No response	7 (4.3)

*Section D*: *Practice of blood donation *	
Have you donated before?	
Yes	36 (22.1)
No	127 (77.9)
How often do you donate?	
<1 time a year	26 (72.2)
1–3 times a year	5 (13.9)
>3 times a year	0 (0.0)
No response	5 (13.9)
Why did you donate?	
A friend or relative needed blood	19 (52.8)
Voluntary	15 (41.7)
Remuneration	1 (2.7)
To know my screening status	1 (2.7)
Will you donate if called upon or reminded to do so?	
Yes	67 (41.1)
No	51 (31.3)
No response	45 (27.6)
Number of those who stated their contact	32 (19.6)
Reasons for non donation by nondonors	
Not approached to donate	32 (25.2)
Unfit to donate	21 (16.5)
Need to donate for friends or relatives in future	25 (19.7)
Fear of needles	8 (6.3)
Fear of knowing my status	4 (3.1)
Religion forbids it	1 (0.8)
Donated blood may be sold	5 (3.9)
No remuneration	1 (0.8)
No response	39 (30.7)
Do you encourage relatives to donate?	
Yes	149 (91.4)
No	7 (4.3)
No response	7 (4.3)

**Table 4:** The association between gender and blood donation.

Gender	Donors (%)	Nondonors	Total
Males	23 (14.5)	46 (28.9)	69 (43.4)
Females	12 (7.5)	78 (49.1)	90 (56.6)

Total	35 (22.0)	124 (78.0)	159 (100)

*χ* ^2^ = 9.1, *P* = 0.003.

**Table 5:** The association between staff category and blood donation.

Category of staff	Donors (%)	Nondonors (%)	Total (%)
Junior	15 (9.4)	54 (33.7)	69 (43.1)
Senior	19 (11.9)	72 (45.0)	91 (56.9)

Total	34 (21.3)	126 (78.7)	160 (100.0)

*χ* ^2^ = 0.017, *P* = 0.895.

**Table 6:** The association between staff level of education and blood donation.

Level of education	Donors (%)	Nondonors (%)	Total (%)
Tertiary	25 (15.8)	97 (61.4)	122 (77.2)
Secondary	7 (4.4)	22 (13.9)	29 (18.3)
Primary	3 (1.9)	3 (1.9)	6 (3.8)
None	0 (0.0)	1 (0.6)	1 (0.6)

Total	35 (22.1)	123 (77.9)	158 (100.0)

Fishers exact test, *P* = 0.334.
